# Hydrogel scaffold with substrate elasticity mimicking physiological-niche promotes proliferation of functional keratinocytes†
†Electronic supplementary information (ESI) available. See DOI: 10.1039/c9ra00781d
‡
‡The raw and the processed data required to reproduce these findings are available from the corresponding author on reasonable request.


**DOI:** 10.1039/c9ra00781d

**Published:** 2019-04-01

**Authors:** Pankaj Mogha, Ankita Srivastava, Sushant Kumar, Sreya Das, Sanjay Kureel, Alka Dwivedi, Atharva Karulkar, Nikita Jain, Abhijeet Sawant, Chitra Nayak, Abhijit Majumder, Rahul Purwar

**Affiliations:** a Department of Biosciences & Bioengineering, Indian Institute of Technology Bombay, Mumbai, Maharashtra 400076, India. Email: purwarrahul@iitb.ac.in; b Department of Chemical Engineering, Indian Institute of Technology Bombay, Mumbai, Maharashtra 400076, India. Email: abhijitm@iitb.ac.in; c Department of Dermatology, B. Y. L Nair Ch. Hospital & T. N. Medical College, Mumbai, Maharashtra 400008, India; d Department of Plastic Surgery, Topiwala National Medical College & B. Y. L. Nair Charitable Hospital, Mumbai, Maharashtra 400008, India

## Abstract

High numbers of autologous human primary keratinocytes (HPKs) are required for patients with burns, wounds and for gene therapy of skin disorders. Although freshly isolated HPKs exhibit a robust regenerative capacity, traditional methodology fails to provide a sufficient number of cells. Here we demonstrated a well characterized, non-cytotoxic and inert hydrogel as a substrate that mimics skin elasticity, which can accelerate proliferation and generate higher numbers of HPKs compared to existing tissue culture plastic (TCP) dishes. More importantly, this novel method was independent of feeder layer or any exogenous pharmaceutical drug. The HPKs from the hydrogel-substrate were functional as demonstrated by wound-healing assay, and the expression of IFN-γ-responsive genes (CXCL10, HLADR). Importantly, gene delivery efficiency by a lentiviral based delivery system was significantly higher in HPKs cultured on hydrogels compared with TCP. In conclusion, our study provides the first evidence that cell-material mechanical interaction is enough to provide a rapid expansion of functional keratinocytes that might be used as autologous grafts for skin disorders.

## Abstract 2

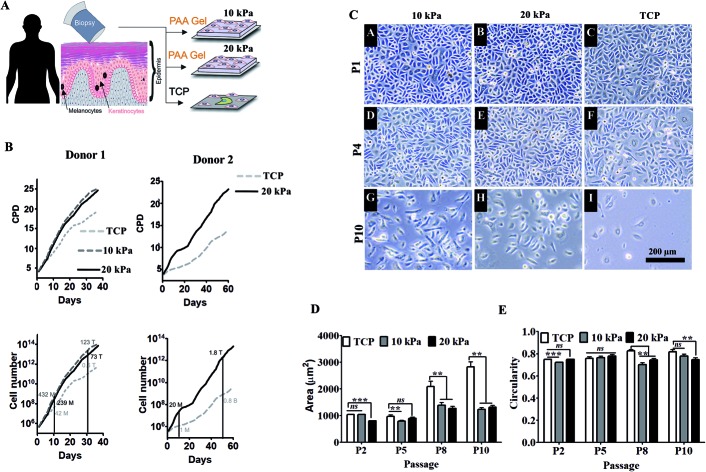
High numbers of autologous human primary keratinocytes (HPKs) are required for patients with burns, wounds and for gene therapy of skin disorders.

## Introduction

Achieving high numbers of functionally intact Human Primary Keratinocytes (HPKs) is critical for the success of skin grafting for multiple skin disorders including gene therapy of epidermolysis bullosa (EB), burns and chronic wounds.^1^,^2^ EB is a genetic skin disorder of connective tissues, which requires full body replacement of the epidermis. There are approximate 19.60 per 1 million live births incidences of EB each year.^3^ Similarly, burns and metabolic wounds especially diabetic ulcers are a serious public health problem and globally almost 11 million people required medical attention for burns.^4^

Skin regeneration using cultured epidermal autografts (CEAs) have been demonstrated highly effective in various skin disorders especially for epidermis replacement in gene therapy of EB and partial thickness wounds. Skin grafting with CEAs does not possess the risk of graft rejection. The traditional method for HPKs expansion done in tissue culture plastic (TCP) dishes is dependent on murine fibroblasts (feeder layer) or dependent on exogenous pharmacological drugs and has been widely used.^5^,^6^ For the treatment of the wound there are various commercial CEAs available. For example, Epicel® (Genzyme Corporation) is supplied as a thin layer of keratinocytes lacking a dermal component. Although this product has proven to be a valuable life-saving treatment option, widespread clinical success has been hindered by technical challenges.^7^ This product is extremely fragile and does not possess barrier function, a property which develops as the multi-layered stratified squamous epithelium matures.^8^ More importantly, Epicel® is available for clinical application only after three to four weeks from the time of skin biopsy necessitating use of other surgical treatment modalities. Furthermore, bacterial contamination and other factors cause high failure rates of Epicel® that are not seen with traditional split-thickness skin grafts.^9^ Similarly another CEA like cellspray is cell suspension and sprayed on damaged area for skin regeneration. Further other commercially available products are Epidex, Myskin, and are based on feeder layer or other exogenous pharmacological drugs. Recently, Rho-inhibitor (Y27632) was demonstrated to promote long-term HPKs culture in absence of feeder layer.^10^ However, for clinical application, it is recommended to avoid xenogeneic materials for transplantation and the cultured keratinocytes must retain the capability to regenerate the epidermis post-transplantation.^11^

To overcome some of these challenges mentioned above, here we describe a well characterized hydrogel based substrate for rapid expansion of functionally intact HPKs and, unlike traditional methodologies; this system neither requires feeder cells (murine or other species) nor exogenous pharmacological drug. In fact, rapid expansion on this substrate is based on the fact that proposed substrate provides optimum mechanical environment by mimicking skin elasticity, a natural habitat for keratinocytes. We further demonstrated that HPKs obtained from our novel method shows delayed senescence and retain higher efficiency of gene delivery, IFN-γ responsive genes, and wound healing abilities.

## Materials and methods

### Substrate preparation

Acrylamide and bis-acrylamide gels were made as previously mentioned.^12^ Briefly, poly-acrylamide gels (PAA) of different stiffness were prepared by cross-linking 40% poly-acrylamide and 2% bis-acrylamide solution in certain ratio, as described. Gel of stiffness 5 kPa was made by mixing 5% acrylamide with 0.15% bis-acrylamide, 10 kPa was made by mixing 10% acrylamide with 0.1% bis-acrylamide and for 20 kPa stiffness 8% acrylamide was mixed with 0.26% bis-acrylamide. Cross-linking of the gels was initiated with 1% ammonium per sulfate and 0.1% of TEMED. The gels were allowed to cross-link for 30 min. Gels were prepared between two glass coverslips, one coated with 3-APTMS ((3-aminopropyl)trimethoxysilane) (Sigma-Aldrich, USA) and the other with octadecyl-trichlorosilane (Sigma-Aldrich, USA). After gelation, the non-adherent coverslip was removed. The gel was coated with type I collagen (25 μg ml^–1^) (Invitrogen, USA) using sulfo-SANPAH (sulfosuccinimidyl 6-(4′-azido-2′-nitrophenylamino)hexanoate) (G-biosciences, USA) based conjugation with UV exposure of 312 nm at 4 °C for overnight.^13^ The gel thickness was modulated by controlling the volume of gel solution placed between the coverslips. Before cell seeding, excess collagen was washed off and the gel was equilibrated with media for one hour. In all the experiments, gels were made on a glass coverslip with the dimension of 22 × 22 mm^2^ and area of approximately 5 cm^2^. For each passage total of 6 such gels were used to give a total area of 30 cm^2^. For TCP, cells were cultured on the 3 wells of a 6-well plate with area of 10 cm^2^ each giving a total area of 30 cm^2^.

### Keratinocyte isolation and culture

Human skin specimens were obtained after cosmetic surgery according to the Declaration of Helsinki Principles and upon approval of Institute Ethical Committee (BYL Nair Charitable hospital, Mumbai and IIT Bombay). Human primary keratinocyte cultures were prepared from discarded samples of cosmetic surgery as described previously.^14^ Briefly, the skin was cut into pieces and incubated overnight at 4 °C in 2.4 U dispase II (Roche, Mannheim, Germany). The next day, the epidermis was separated from the dermis and placed for 20 minutes at 37 °C in trypsin–EDTA solution (0.25%) (Himedia, India). After stopping the trypsin reaction by addition of neutralizing media (DMEM + 10% FBS, Gibco) the cell suspension was filtered through cell strainer (40 μm) and washed two times in neutralizing media (DMEM + 10% FBS. Gibco). The single-cell suspension of HPKs was incubated in serum-free Keratinocyte Growth Medium EpiLife (Gibco; 0.06 mM Ca^++^) and supplemented with EpiLife defined growth supplements (Human keratinocyte growth supplement, Gibco) at 37 °C in a humidified atmosphere containing 5% CO_2_.

The cells were seeded onto the substrates (gels and plastic) at the seeding density of 7000 cells per cm^2^ (total number of cells seeded for gels and plastic was around 0.21 million). Once the cells had reached the confluency of 80% the cells were trypsinized and seeded onto the fresh set of substrates (gel and plastic) with the same seeding density of 7000 cells per cm^2^.

### Cell population doubling calculation

The cells were seeded onto the collagen coated hydrogel scaffold system of control (collagen coated cell culture dish) with the seeding density of 7000 cells per cm^2^. After 4 hours, imaging was done using EVOS XL Core cell imaging system (ThermoFisher Scientific, USA) to quantify the number of cells adhered onto the gels and TCP. Cell counting was performed using the ImageJ software. This was obtained by dividing the number of cells per frame by frame area followed by multiplication with the total area of the substrate. Cell number was again quantified at ∼80% confluency in order to calculate the population doubling. The population doubling was calculated by using the eqn (1).

1 *N* = *N*_o_ × 2^*n*^

where *N* is total number of cells obtained after *n* number of cellular division for *N*_o_ number of cells which were present initially. *n* gives the population doubling (PD) of the cells which was calculated at every passage which was added to the PD of the previous passage to obtain the cumulative population doubling (CPD).

### BrdU staining

The bromodeoxyuridine (BrdU) incorporation assay was used to identify proliferating cells. Cells seeded on gels and TCP were incubated with medium containing BrdU labeling agent (dilution 1 : 200; Abcam) for 24 hours. Cells were then fixed with 4% paraformaldehyde, denatured by 2 M HCl for 10 minutes, permeabilized with 0.5% Triton-X-100 (Sigma Aldrich) for 30 minutes and blocked with 1% bovine serum albumin. Anti-BrdU antibody (dilution 1 : 200, Thermo-scientific, USA) was added to the BrdU labeled cells and was incubated overnight at 4 °C. After several washes with PBS, secondary antibody Alexa Fluor®568 Rabbit anti-mouse (dilution 1 : 1000, Thermo-scientific, USA) was added along with a nuclear counterstain (Hoechst, 33 342, Thermo-scientific, USA). Labeled cells were imaged using EVOS FL Auto (Life Technologies, USA) at 10× magnification.

### β-gal staining

The HPKs were obtained from gel and TCP was seeded on the collagen coated glass coverslips. After 24 hours, cells were stained with β-galactosidase (β-gal) as explained previously.^15^ Briefly, the cells were washed thrice with PBS and fixed with the 4% paraformaldehyde for 10 minutes. The fixative buffer was removed and cells were washed again thrice with PBS. This was followed by overnight incubation in the staining solution (containing X-gal) at 37 °C without CO_2_. The cells were then washed and imaged under 10× magnification in EVOS FL Auto inverted fluorescence microscope.

### Immunofluorescence

HPKs were seeded on collagen coated 18 mm glass coverslips, the cells were washed thrice with warm 1× PBS. The cells were then permeabilized with an ice cold 1 : 1 solution of 4% paraformaldehyde and 0.5% Triton X-100 for 1 min on ice. The cells were washed with cold 1× PBS and incubated for 5 minutes with cold 4% paraformaldehyde on ice. Post 3 washes with cold 1× PBS and incubating with blocking buffer (1.5% BSA in 0.5% Triton X-100) for 30 minutes on ice, the samples were incubated at 4 °C overnight in anti-vinculin primary antibody, followed by Alexa Fluor 488 conjugated secondary antibody (1 : 1000) and Alexa Fluor 647 conjugated phalloidin (1 : 200) at room temperature for 2 hours. Images were captured using an LSM 780 confocal microscope (Zeiss) under 63× magnification. Number and area of the focal adhesions was quantified by ImageJ software.

### mRNA isolation, cDNA synthesis and qRT-PCR

RNA isolation from HPKs derived from early passage P-2 and late passages P-6 and P-11 from 10 kPa, 20 kPa and TCP were performed using RNeasyPlus Mini Kit (Qiagen, Hilden, Germany). First strand cDNA synthesis was performed using Quantitect Reverse Transcription kit (Qiagen, Hilden, Germany) and quantitative Real-Time PCR was performed using SYBR® Premix Ex Taq™ II (Tli RNase H Plus) (Takara, Otsu, Japan) by StepOnePlus™ Real-time PCR System (Applied Biosystems, California, USA). Target genes were amplified (initial denaturation to activate polymerase activity at 95 °C for 30 seconds, denaturation at 95 °C for 5 seconds, anneal/extension at 60 °C for 30 seconds) using filaggrin (FLG), involucrin (INV), keratin 19 (KRT19), keratin 14 (KRT14) and GAPDH pre-designed primers obtained Kicqstart (Sigma-Aldrich, USA). Quantification of the relative gene expression was performed using StepOne™ Software v2.3.

### Transduction of primary keratinocytes using lentiviral vector

Lentiviral particles were generated by using 3^rd^ generation lentiviral system. Four plasmids (packaging: pMD2G, envelop: pMDLg/pRRE and pRSV-Rev and target vector: pHIV-EGFP) were procured from Addgene, (Plasmid repository, USA). Lentiviral packaging cell line (Lenti-X 293T cells) procured from Clontech (USA). Lenti-X 293T cells were transfected with Lipofectamine 3000 (Invitrogen, USA) and lentivirus particles were produced with titer of 2.8 × 10^5^ TU per ml. Next, 5 × 10^5^ HPKs obtained from TCP and 20 kPa gel were seeded in 24-well plate in 500 μl of keratinocyte growth medium. Next day, lentivirus particles (MOI-0.5) were added in the presence of polybrene and incubated for 24 h. One well was kept as a control without virus addition. Post 24 h, 1 ml keratinocytes growth medium was added in each well and incubated for next 48 h. Percentage transduction efficiency was measured by GFP fluorescent intensity in BD FACSVerse flow cytometer.

### Quantification of IFN-γ responsive genes (HLA-DR and CXCL-10)

HPKs (2 × 10^5^/0.5 ml) were cultured in 24-well plate in presence of IFN-γ (20 ng ml^–1^) at 37 °C, 5% CO_2_. After 24 hours of incubation, cells were trypsinized and washed twice with staining buffer. HPKs were re-suspended in 100 μl staining buffer and stained with PerCP-Cy5.5-conjugated anti-HLA-DR mAb (clone: G46-6) for 40 min on ice. After incubation, HPKs were washed twice and resuspended in staining buffer and acquired in BD FACSVerse flow cytometer and analyzed using BD FAC Suite software (BD Biosciences).

For CXCL10 quantification, HPKs (2 × 10^5^/0.5 ml) were cultured in 24-well plate in presence of IFN-γ (20 ng ml^–1^) at 37 °C, 5% CO_2_. After 24 h of incubation, cell free supernatant was collected. CXCL10 was detected by ELISA Max™ Deluxe set (Biolegend, San Diego, CA) according to manufacturer's instructions and the concentration was determined by absorbance at 450 nm measured using Multiskan Go (Thermo Fisher Scientific, USA)

### Scratch assay

HPKs obtained from TCP, 10 kPa and 20 kPa were grown on a tissue-culture treated 12-well plate till a 100% confluent monolayer was obtained. A 200 μl sterile tip was used to scratch off the cells to create a cell free area. Cells were washed gently multiple times to remove all the de-adhered cells. Incomplete keratinocyte media (-BPE, -EGF) was used in all the conditions to minimize cell proliferation. Epidermal growth factor (EGF, 2 ng ml^–1^) was used as a positive control. Cells were imaged under 10× magnification at 0 hours, 24 hours, 48 hours, and 72 hours or until 100% closure was seen. The cell free area was calculated using the ImageJ software. The percent (%) of wound closure was calculated as per following formula:
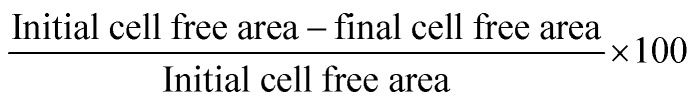


### Statistical analysis

Comparative data were analyzed by using, *t*-test, and paired *t*-test (paired data are depicted). The software used to perform the statistical analysis was Prism 7.02. Mean values ± standard errors of mean are depicted. In the figures, *stands for a *P*-value <0.05, **P < 0.02, and ***P < 0.01.

## Results

### Hydrogel substrates with elasticity similar to human skin provide rapid human primary keratinocyte (HPKs) expansion

**Fig. 1:**
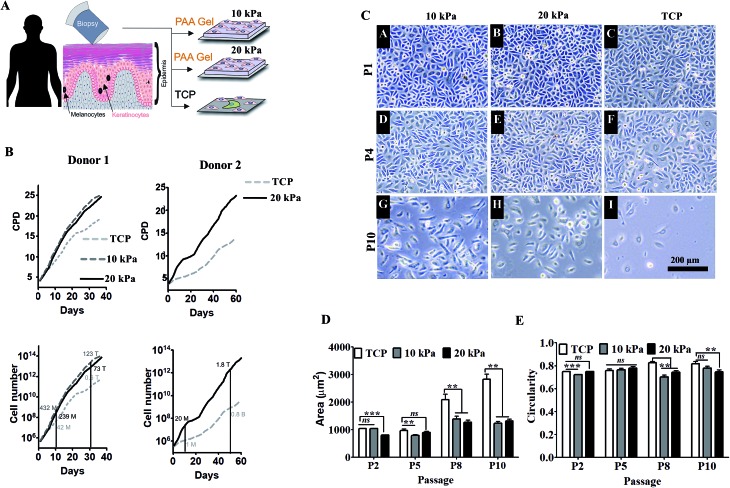
Hydrogel substrates with elasticity similar to human skin provide rapid human primary keratinocytes (HPKs) expansion: human primary keratinocytes (HPKs) were expanded for several weeks either in hydrogels or using standard tissue culture plastic dishes (TCP). HPKs expansion, proliferation rate and morphology were examined. (A) The CPD value for two donors across the duration of cell culture. (B) The extrapolated values of the cell number obtained after culturing the cells (seeding density: 0.3 million) onto the different substrate stiffness. (C) Analysis of cellular morphology at P1, P4, and P10 on stiffness of 10 kPa, 20 kPa, and TCP (scale 200 μm). (D and E) The quantitative analysis of cell area and circularity on different stiffness across different passage.

We prepared inert hydrogel substrates made of polyacrylamide gels, elasticity of which was tuned by varying cross-linking density to match skin elasticity (5–20 kPa) as described in materials and methods. These well-defined and tunable gels were characterized previously for optimal expansion and differentiation of mesenchymal stem cells (ESI Fig. 1A and B and our unpublished data†). For cell adhesion, gels were coated with collagen I, which is also the major component of basement membrane of epidermis. Human primary keratinocytes were obtained from healthy subjects and were expanded on collagen coated hydrogel with variable substrate elasticity (5 kPa, 10 kPa, or 20 kPa) for over 1–2 months as shown in the schematic (Fig. 1A). HPKs cultured on substrate with 5 kPa (softer matrix) did not survive (ESI Fig. 1C†). However, as depicted in Fig. 1B, HPKs cultured on substrate with 10 kPa and 20 kPa elasticity showed rapid expansion compared with control (collagen coated TCP dish: 1 GPa) and showed shorter doubling time, resulting into significantly higher cumulative population doubling (CPD) and high numbers of HPKs compared to that on control (Fig. 1B). This data suggests that substrates with optimum stiffness support rapid expansion of HPKs without the need of feeder layer or any exogenous pharmacological drugs.

Not just the proliferation rate, softer substrates also help to maintain cellular morphology as shown in Fig. 1C. Cell spreading increased with passage on plastic. They became highly elongated taking up morphology of the differentiated cells. However, cells were smaller in softer gels compared with control in all passages (Fig. 1D). On the other hand, circularity was found to be similar in both conditions (10/20 kPa and control, Fig. 1E), indicating that optimal substrate elasticity is critical for retaining their cobblestone morphology and shape.

### Substrates with physiological stiffness promotes self-renewal and delays senescence and spontaneous differentiation

**Fig. 2:**
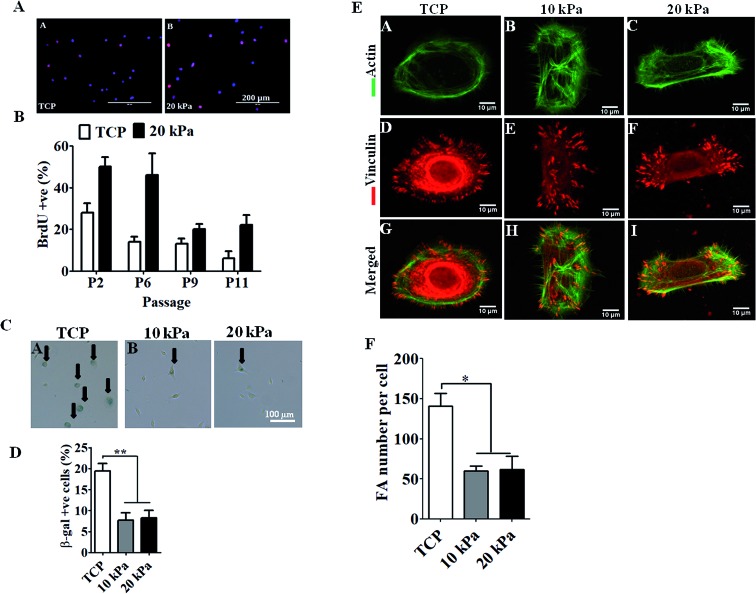
Substrates with physiological stiffness promotes self-renewal and delays senescence. (A) The representative images (left: TCP, right: 20 kPa) shows the cells going into S-phase of cell division through BrdU incorporation, BrdU positive cells are shown with magenta and nucleus in blue colour (scale 200 μm). (B) Bar graph shows the quantification for percentage of BrdU positive cells on respective stiffness (no. of cells >39) (C and D) representative images (C) and cumulative data depicting percentage of β-gal positive cells (D) for identification and quantification of β-gal positive cells respectively (scale 100 μm). (E and F) Quantification of focal adhesion by actin, and vinculin staining of HPKs. Representative images (E) of actin (green, top row), vinculin (red, middle row) and merged (bottom row) are depicted and quantification of the focal adhesion numbers per cell on different stiffness (F) are depicted as mean ± SEM (no. of cells ≥5).

Next we examined the proliferative capacity of HPKs by quantifying the frequency of BrdU positive cells. HPKs were obtained at various passages from either softer gel or control. As depicted in Fig. 2A and B, high numbers of BrdU incorporated HPKs were present on softer substrate (10 kPa/20 kPa) compared to control. Next, we examined the percentage population of senescent cells by staining with β-gal^+^ to examine the effect of extensive expansion on gels of elasticity 10 kPa, 20 kPa, and TCP (Fig. 2C and D). Total numbers of cells analyzed were at least 200 for each condition from passage 6. There were fewer β-gal^+^ cells when HPKs were expanded on softer gel compared to control. Collectively these data suggest that softer gel supports prolonged HPKs proliferation and inhibit senescence during extensive expansion.

Next we checked the differentiation states of the cells originating from the gels and TCP during the extensive expansion. We examined the mRNA expression profile of early (KRT19), late (KRT10) and terminal differentiation markers (involucrin and filaggrin) on cells expanded on gel substrates of (10–20 kPa) and controls for early and later passages. As shown in ESI Fig. 2,† expression of early differentiation marker (KRT19) increased in cells expanded from passages 2 to 11 in control as well as in cells obtained from 10 kPa and 20 kPa scaffolds, expression of late differentiation markers (KRT10) and terminal differentiation markers (filaggrin and involucrin) was downregulated in HPKs expanded on gels compared to controls.

### Reduced focal adhesion complexes of HPKs with softer matrix compared with control

Next we examined how tuning the substrate elasticity impacts the cell matrix interaction. Focal adhesion complexes are the first molecules to sense the substrate elasticity and to transduce the mechano-signals from outside to inside of the cell. HPKs expanded on softer gel and control was stained for actin, and vinculin and the focal adhesion number per cell was quantified. As depicted in Fig. 2E and F, HPKs from 10 kPa and 20 kPa showed fewer numbers of focal adhesion compared to control, suggesting different level of physical interaction of HPKs with gel and plastic.

### Human primary keratinocytes (HPKs) expanded on 10 kPa and 20 kPa gel respond to IFN-γ-responsive genes (HLA DR and CXCL10)

**Fig. 3:**
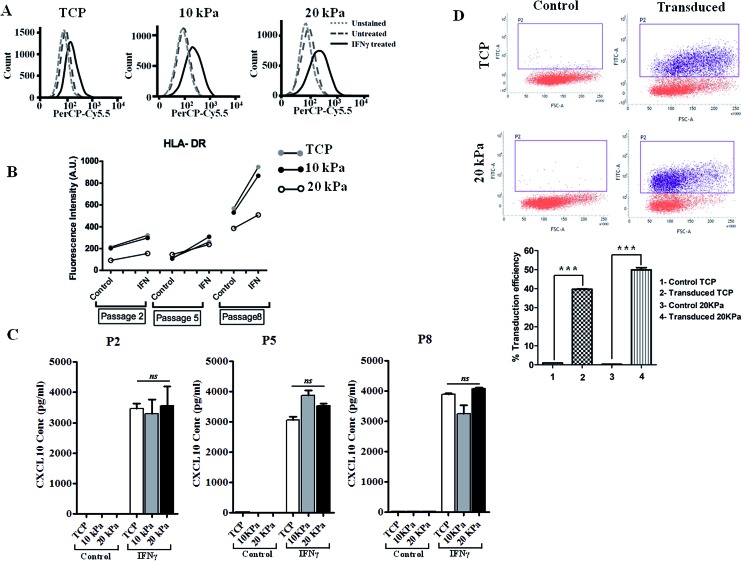
Human primary keratinocytes (HPKs) expanded on 10 kPa and 20 kPa gel respond to IFN-γ-responsive genes (HLA DR and CXCL10) and suitable for efficient transduction of genes by lentiviral mediated gene delivery vehicle. (A–C) HPKs were harvested from softer substrate and controls at early stage and late passages and cultured in presence of IFN-γ and HLA-DR expression was analyzed by flowcytometry (A and B) and CXCL10 secretion was quantified in cell free supernatant by ELISA (C). Dotted lines in histogram represent unstained control. Dashed lines and black lines represent staining of un-stimulated and IFN-γ-treated cells respectively. Mean ± SEM is given. (D) Lentiviral transduction of HPKs grown on TCP and 20 kPa, 5 × 105 HPKs grown on TCP and 20 kPa transduced with GFP lentiviral particles with MOI-0.56, representative dot plots depict GFP intensity showing percentage transduction efficiency and bar graphs represent the transduction efficiency from triplicates.

IFN-γ is primarily a Th1 cell cytokine and present in human skin in many skin inflammatory disorders and impact keratinocyte proliferation, apoptosis, migration, *etc.*^16^,^17^ To examine if HPKs obtained from hydrogel are functionally responsive to immune effector molecules such as IFN-γ, HPKs expanded on 10 kPa and 20 kPa gel and control at early and late passages were cultured in presence of IFN-γ and surface expression of HLA DR was examined by staining with HLA DR specific mAb and were analyzed by flowcytometry. IFN-γ increased the HLA-DR expression on keratinocytes expanded on 10 kPa, 20 kPa and controls in both early (P-2) and late passages (P-5 to P-8) (Fig. 3A and B).

Further, CXCL10 (IP10), a chemokine responsible for monocyte and T cell recruitment to the skin in various skin inflammatory conditions^18^,^19^ was also examined. HPKs from softer gel and control from early and late passage was stimulated with IFN-γ and 24 h later cell-free supernatant was collected and CXCL10 was quantified using CXCL10 specific ELISA. IFN-γ treatment of HPKs significantly upregulated the CXCL10 secretion in HPKs of 10 kPa, 20 kPa gels and control conditions in both early and late passages (Fig. 3C), suggesting that HPKs obtained from hydrogel based scaffold system are IFN-γ-responsive.

### HPKs expanded in soft substrate are suitable for gene therapy

The lentiviral/retroviral mediated gene delivery system is considered one of the most used gene delivery system in the clinic for stable transduction of genes including in epidermolysis bullosa and other genetic disorders. Gene transduction efficiency of HPKs expanded on 20 kPa and tissue culture treated plate (TCP) were examined and compared with control. Lentiviral vector was produced as described in method section using 3^rd^ generation plasmids and packaging cell line. HPKs from 20 kPa substrate and TCP were infected with lentiviral particles and as depicted in Fig. 3D demonstrate that HPKs expanded on 20 kPa substrate are suitable for gene delivery using lentiviral particle. Interestingly, gene transduction efficiency of HPKs expanded in 20 kPa substrate was significantly higher compared to TCP (Fig. 3D). This data suggest that HPKs expanded using this method is suitable for gene therapy application.

### HPKs expanded on hydrogels posses better would healing capacity compared to control upon extensive expansion

**Fig. 4:**
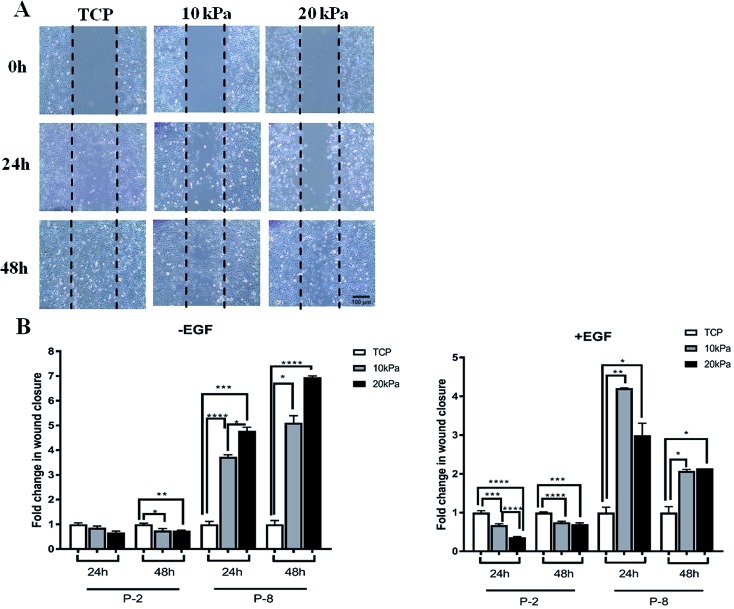
HPKs expanded on hydrogels possess better would healing capacity compared to control upon extensive expansion. (A) The representative images depict wound closure by HPKs obtained from TCP, 10 kPa and 20 kPa of P-2 under 10× magnification in the presence of EGF. The black dashed line marks the wound front and cell migration is shown till 48 h. (B) Graph represents the fold change of the percentage of wound closure in 24 h and 48 h, normalized to TCP of the corresponding time point. The left bar graph (without epidermal growth factor: – EGF) depicts the retention of the inherent migratory nature of keratinocytes even at P-8. The right bar graph (plus EGF: +EGF) represents migratory responsiveness of the cells towards EGF (2 ng ml^–1^). At higher passage, significantly higher migration was observed in cells obtained from 10 kPa and 20 kPa than TCP with and without EGF. Bar graph represents mean ± SEM.

Clinically it is critical for HPKs to retain wound healing abilities upon extensive proliferation. To examine if HPKs retain this potential upon extensive proliferation, we did a scratch assay of HPKs of early and late passages obtained from hydrogels (10 and 20 kPa) and control. While motility (wound healing capacity) of HPKs obtained from gels and control took similar time for complete wound closure in lower passages, surprisingly, migration of HPKs of later passages obtained from softer gel (10 kPa and 20 kPa) was significantly faster in wound closure compared to control. Fig. 4A shows the representative images of the wound generated on TCP, 10 kPa, and 20 kPa gels, for 0 hours, 24 hours, and 48 hours time points. Quantitative analysis of the wound closure across different substrates and time points revealed that HPKs obtained from softer gel are able to migrate and functional (Fig. 4B). More importantly, softer hydrogel supports HPKs retaining their wound healing potential even in later passages which gets compromised for cells cultured on plastic (Fig. 4B).

## Discussion

In this study, we have provided the first ever evidence of rapid expansion of human primary keratinocyte using a well-characterized polyacrylamide based substrate with defined elasticity. Interestingly, this novel method of keratinocyte expansion does not require exogenous growth factors or feeder layer. Further, HPKs expanded in the 10–20 kPa gel were suitable for wound healing, responsiveness to immune-effector molecules and suitable for gene therapy.

Given the extensive demand of high numbers of HPKs for wound healing, scar reduction and gene therapy, skin grafts, and the requirement of alternatives to animal testing there is a need of developing novel method for human primary keratinocyte expansion without perturbing their function. Current methods require feeder layer or exogenous factors for rapid expansion and some of them do not provide large numbers of HPKs in short duration without perturbing their function.^20^ Here we investigated the roles of modifying the mechanical environment of HPKs expansion. One of the key regulators of mechanical cues is substrate elasticity of the matrix. The polyacrylamide gel system are well characterized and previously used for stem cell regeneration and differentiation. It was demonstrated that elasticity of gel impacts the stem cell lineage specific differentiation. Stem cells cultured on substrate that mimic the elasticity of muscle (*E*_muscle_: 10 kPa) was suitable for myogenic differentiation and matrix mimicking bone elasticity (*E*_bone_: 34 kPa) differentiated the stem cells into osteoblast without the need of exogenous differentiation factors, suggesting that physical forces by which stem cell senses the matrix provide the mechanical signaling requires for stem cell lineage specific commitment.^21^ There has been ample evidence to support that the mechanical properties of culture surface can influence the adhesion, proliferation and differentiation of stem cells and fibroblasts.^21^–^23^ Similarly, myogenic stem cells cultured on soft hydrogel substrates that mimic the elasticity of muscle (12 kPa) self-renew *in vitro* and contribute extensively to muscle regeneration when subsequently transplanted into mice.^24^ However there are no studies examining the impact of substrate elasticity of matrix on the HPKs expansion.

There are several studies reported highly variable skin elasticity ranging from 0.1 kPa to 10 kPa;^25^ 23–70 MPa ;^26^ 18–57 MPa (ref. 27) and has been shown to influenced by the part of the body and the age of the donor.^25^ Because skin is one of the largest organ in the human body,^28^ elasticity of various part of the skin differ significantly due to multi-layer epidermis, thickness of dermis, underneath muscle, and hydration level. To address some of these problems, recently tribometer device was used to demonstrate to measure the elasticity of various skin layer and mathematically model the elasticity of dermis (35 kPa), hypodermis (2 kPa) and muscle (80 kPa).^29^ Of course these elastic modulus are not fixed and may vary depending on the resting or functional state of the skin. As mentioned earlier matrix stiffness influence the cell's properties, the expansion of the cells was done with varying substrate stiffness of poly-acrylamide gels. The cells did not survive beyond passage 3 when cultured on stiffness of 5 kPa (ESI Fig. 1†). On the other hand, when cultured on a slightly stiffer matrix (10 kPa and 20 kPa) cells showed survival and showed higher proliferation as compared to TCP (Fig. 1) which has also been shown with PDMS.^30^ This concludes that stiffness not only plays a critical role in regulating the proliferation but is also involved in cell survival.

The cells from the gels shower higher proliferative capabilities as depicted from the CPD values obtained from two healthy independent donors. The cells being cultured on the gels always showed higher CPD compared to the TCP at any given time. The proliferation of the cells though were different among the different patients but TCP always remained less than that of the gel, thus showed stiffness dependence despite of change in the source.

Researchers have made keratinocytes expanded for very long duration by inhibiting rho-associated coiled-coil containing protein kinase (ROCK) activity with Y27632. The result shows that inhibition of ROCK increases telomerase activity of the cells resulting into stopping senescence.^31^ These cells did not show any kind of genetic instability, and managed to maintain the ability to differentiate and produce the 3D skin. Also higher activity of telomerase has been shown to induce tumour and are constructively expressed in cancer cells.^32^ To make any cell constitutively expressive towards any gene the cells are required to be transfected with the help of viruses^33^ which renders the cells for clinical application. It has also been shown that low concentration of TGF-β1 influence the long term expansion of keratinocytes,^34^,^35^ but the expression of TGF-β1 has been shown to be associated with skin tumours.^36^ Thus cell expansion with gels provides the best suitable method for expansion of the cells for longer duration. In addition to proliferation, the effect of stiffness on migration of HPKs was studied after prolonged passaging on TCP and biomaterials (PAA gels). Interestingly, in HPKs at early passages, diminished differences were observed in cell migratory behavior when comparing between 10 kPa and 20 kPa gels as well as rigid TCP control (Fig. 4B, -EGF). However at late passage, which is a result of prolonged culturing of cells on compliant substrate, HPKs obtained from gels migrates faster in wound closure compared to the control. This observation indicate that some kind of cellular adaptation have occurred upon prolonged passaging on compliant substrates. Interestingly, cells also seemed to “remember” the stiffness to which they have been pre-conditioned when passaged above a threshold period of time exhibiting ‘mechanical memory’. However when comparing migratory behavior of cells from 10 kPa and 20 kPa in the presence of exogenous EGF, we found cells at 10 kPa migrated faster than cells obtained from 20 kPa gels (Fig. 4B, +EGF). This observation was interesting and provided evidence that mechanical forces might play a key role in cell motility. It is possible that cells sense and respond by virtue of its mechanical environment and acquire the stiffness sensitive phenotype based on integrin expression. Studies have shown that integrin α-5 expression increases 5-fold on a 10 kPa stiffness compared to 1 kPa in cancer cells and fibroblast.^37^,^38^ This indicates that for the expression of integrins, 10 kPa is the most biologically relevant stiffness. Integrin play crucial role in cell motility *via* positive association with EGF receptor and cells move faster when exogenous EGF is added.^39^ This could possibly explain the differential movement of cells in 10 kPa and 20 kPa gels in the presence of EGF. This data indicates that cell motility has biphasic dependency on substrate stiffness.

Further, the gels helped in maintenance of their native state shown by higher expression of KRT19 and KRT10 and lower expression of filaggrin and involucrin. Thus our gel system provides many times higher cell number without losing the properties of stem cells as compared to traditional cell culture system.

## Contributions

A. M. and R. P. conceptualized and designed the experiments, methodology, and supervised the project. C. N. and A. G. provided the tissue samples for keratinocyte isolation. S. K. K. optimized the material preparation protocol, P. M. did material preparation. A. S. isolated HPKs, cultured and maintained the cells. P. M., A. S. and S. K. did CPD and morphology image acquisition and analysis. P. M. performed BrdU, beta-gal, vinculin staining, imaging, and analysis. A. S. and S. D. performed RT-PCR. S. D. performed and analyzed scratch assay. S. K., N. J. and A. S. performed and analyzed FACS and ELISA assay. A. D. and A. K. performed keratinocyte transfection. P. M., A. S., S. K., S. D., A. D. and A. K. prepared the figures. P. M., A. S., S. K., N. J., S. D., A. D., A. K., A. M., and R. P. interpreted the results and wrote the manuscript. P. M., A. S. and S. K. contributed equally for the paper to be joint first authors.

## Conflicts of interest

Authors declare no competing interests.

## Supplementary Material

Supplementary information

## References

[cit1] Dai N. T., Williamson M. R., Khammo N., Adams E. F., Coombes A. G. (2004). Biomaterials.

[cit2] Groeber F., Holeiter M., Hampel M., Hinderer S., Schenke-Layland K. (2011). Adv. Drug Delivery Rev..

[cit3] Fine J. D. (2016). JAMA Dermatol..

[cit4] Peck M. D. (2011). Burns.

[cit5] Kim D. S., Cho H. J., Choi H. R., Kwon S. B., Park K. C. (2004). Cell. Mol. Life Sci..

[cit6] Rheinwald J. G., Green H. (1975). Cell.

[cit7] Atiyeh B. S., Costagliola M. (2007). Burns.

[cit8] Nemes Z., Steinert P. M. (1999). Exp. Mol. Med..

[cit9] Cairns B. A., deSerres S., Peterson H. D., Meyer A. A. (1993). Arch. Surg..

[cit10] Llames S., Garcia-Perez E., Meana A., Larcher F., del Rio M. (2015). Tissue Eng., Part B.

[cit11] Ter Horst B., Chouhan G., Moiemen N. S., Grover L. M. (2018). Adv. Drug Delivery Rev..

[cit12] Tse J. R. Engler A. J., Curr. Protoc. Cell Biol., 2010, 10.16.1–10.16.16, , chapter 10, unit 10.16. 20521229 10.1002/0471143030.cb1016s47

[cit13] Venugopal B., Mogha P., Dhawan J., Majumder A. (2018). Biomater. Sci..

[cit14] Wittmann M., Purwar R., Hartmann C., Gutzmer R., Werfel T. (2005). J. Invest. Dermatol..

[cit15] Debacq-Chainiaux F., Erusalimsky J. D., Campisi J., Toussaint O. (2009). Nat. Protoc..

[cit16] Purwar R., Wittmann M., Zwirner J., Oppermann M., Kracht M., Dittrich-Breiholz O., Gutzmer R., Werfel T. (2006). J. Immunol..

[cit17] Das S., Srinivasan S., Srivastava A., Kumar S., Das G., Das S., Dwivedi A., Karulkar A., Makkad K., Bilala R., Gupta A., Sawant A., Nayak C., Tayalia P., Purwar R. (2019). J. Immunol..

[cit18] Richmond J. M., Bangari D. S., Essien K. I., Currimbhoy S. D., Groom J. R., Pandya A. G., Youd M. E., Luster A. D., Harris J. E. (2017). J. Invest. Dermatol..

[cit19] Petrovic-Djergovic D., Popovic M., Chittiprol S., Cortado H., Ransom R. F., Partida-Sanchez S. (2015). Clin. Exp. Immunol..

[cit20] Breidahl A. F., Judson R. T., Clunie G. J. (1989). Aust. N. Z. J. Surg..

[cit21] Engler A. J., Sen S., Sweeney H. L., Discher D. E. (2006). Cell.

[cit22] Balaban N. Q., Schwarz U. S., Riveline D., Goichberg P., Tzur G., Sabanay I., Mahalu D., Safran S., Bershadsky A., Addadi L., Geiger B. (2001). Nat. Cell Biol..

[cit23] Choquet D., Felsenfeld D. P., Sheetz M. P. (1997). Cell.

[cit24] Gilbert P. M., Havenstrite K. L., Magnusson K. E., Sacco A., Leonardi N. A., Kraft P., Nguyen N. K., Thrun S., Lutolf M. P., Blau H. M. (2010). Science.

[cit25] Achterberg V. F., Buscemi L., Diekmann H., Smith-Clerc J., Schwengler H., Meister J. J., Wenck H., Gallinat S., Hinz B. (2014). J. Invest. Dermatol..

[cit26] Gaikwad R. M., Vasilyev S. I., Datta S., Sokolov I. (2010). Skin Res. Technol..

[cit27] Grahame R., Holt P. J. (1969). Gerontologia.

[cit28] Grice E. A., Segre J. A. (2011). Nat. Rev. Microbiol..

[cit29] Pailler-Mattei C., Bec S., Zahouani H. (2008). Med. Eng. Phys..

[cit30] Gupta P., Hari Narayana S. N. G., Kasiviswanathan U., Agarwal T., Senthilguru K., Mukhopadhyay D., Pal K., Giri S., Maiti T. K., Banerjee I. (2016). RSC Adv..

[cit31] Chapman S., McDermott D. H., Shen K., Jang M. K., McBride A. A. (2014). Stem Cell Res. Ther..

[cit32] Tomas-Loba A., Flores I., Fernandez-Marcos P. J., Cayuela M. L., Maraver A., Tejera A., Borras C., Matheu A., Klatt P., Flores J. M., Vina J., Serrano M., Blasco M. A. (2008). Cell.

[cit33] Bocker W., Yin Z., Drosse I., Haasters F., Rossmann O., Wierer M., Popov C., Locher M., Mutschler W., Docheva D., Schieker M. (2008). J. Cell. Mol. Med..

[cit34] Fortunel N. O., Hatzfeld J. A., Rosemary P. A., Ferraris C., Monier M. N., Haydont V., Longuet J., Brethon B., Lim B., Castiel I., Schmidt R., Hatzfeld A. (2003). J. Cell Sci..

[cit35] Purwar R., Werfel T., Wittmann M. (2007). J. Invest. Dermatol..

[cit36] Lange D., Persson U., Wollina U., ten Dijke P., Castelli E., Heldin C. H., Funa K. (1999). Int. J. Oncol..

[cit37] Leuschner U., Kurtz W. (1987). Lancet.

[cit38] Yeung T., Georges P. C., Flanagan L. A., Marg B., Ortiz M., Funaki M., Zahir N., Ming W., Weaver V., Janmey P. A. (2005). Cell Motil. Cytoskeleton.

[cit39] Schwartz A. D., Hall C. L., Barney L. E., Babbitt C. C., Peyton S. R. (2018). Biomaterials.

