# Supplementary material for: Hydrogel scaffold with substrate elasticity mimicking physiological-niche promotes proliferation of functional keratinocytes
Source: RSC Adv. 2019 Apr 1;9(18):10174–83. doi: 10.1039/c9ra00781d (PMC6592153; doi:10.1039/c9ra00781d)
Supplement: RA-009-C9RA00781D-s001 [file RA-009-C9RA00781D-s001.pdf]

Electronic Supplementary Material (ESI) for RSC Advances.
This journal is © The Royal Society of Chemistry 2019

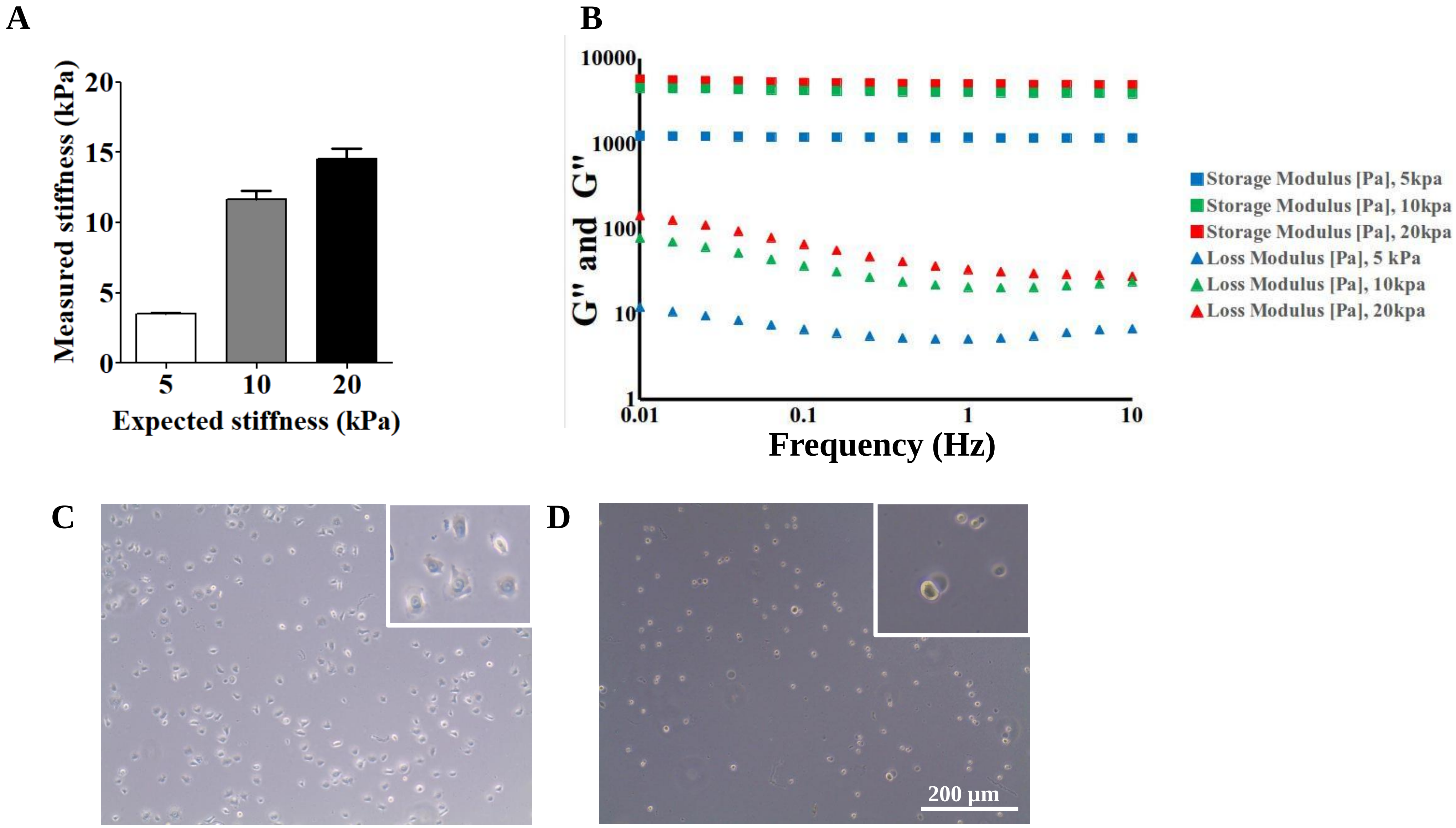

Supplementary Figure 1: Characterization of tunable inert hydrogel substrates made of polyacrylamide gels with varying elasticity and HPKs grown in 5-kPa gel: A) Shows the bar graph for the Young's Modulus of the gels indicating their elasticity **B)** Shows the storage and loss modulus of the gels measured by using Anton-Paar Rheometer. The frequency sweep was preceded by a 25 minute time sweep to allow for gels to solidify. Frequency tests were performed with frequency decreasing from 628 to 0.628 rad/s at a strain of 1 %. **C)** Shows the phase image of the cells of P3 seeded on the stiffness of 5 kPa with a seeding density of 7000 cells per cm^2. The zoomed section is shown in the inset, where cells are attached to the matrix and spread. **D)** Shows the phase image of the cells after 5 days of cell culture. The inset shows the dead cells attached to the substrate. Scale is 200 µm.

Supplementary Figure 1

(A) Expression of early differentiation marker

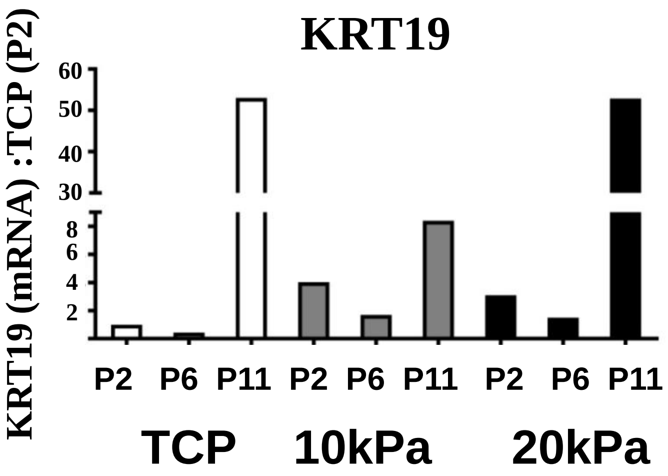

(B) Expression of late differentiation marker

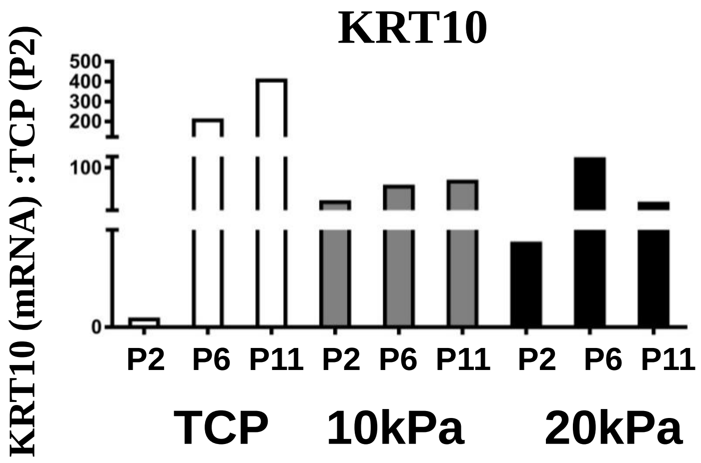

(C) Expression of terminal differentiation markers

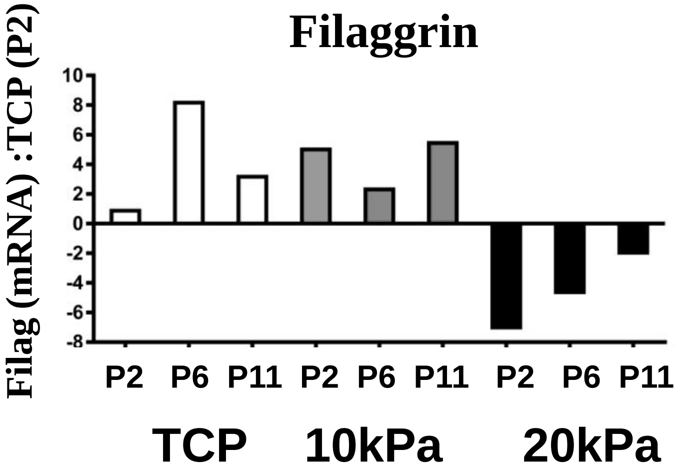

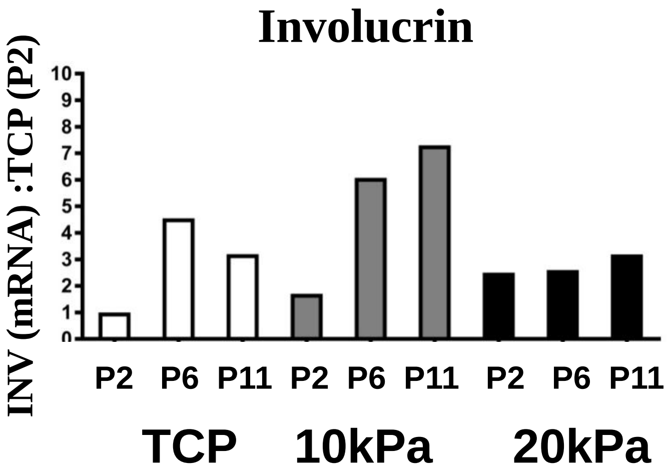

Supplementary Figure 2

Expression levels of Differentiation markers in Primary Human keratinocytes on a variety of substrate stiffness was probed using real-time RT-PCR. Changes of Substrate elasticity and passage dependent mRNA expression of differentiation marker keratin 19 (KRT 19), Keratin 10 (KRT10), Fillaggrin (FLG), Involucrin IV of primary human Keratinocytes seeded on 10 kPa, 20 kPa and TCP at P2, P6, and P11. Genes were normalized to individual GAPDH and TCP P-2 used as reference control. Results are mean±SD, n=3

Supplementary Figure 2

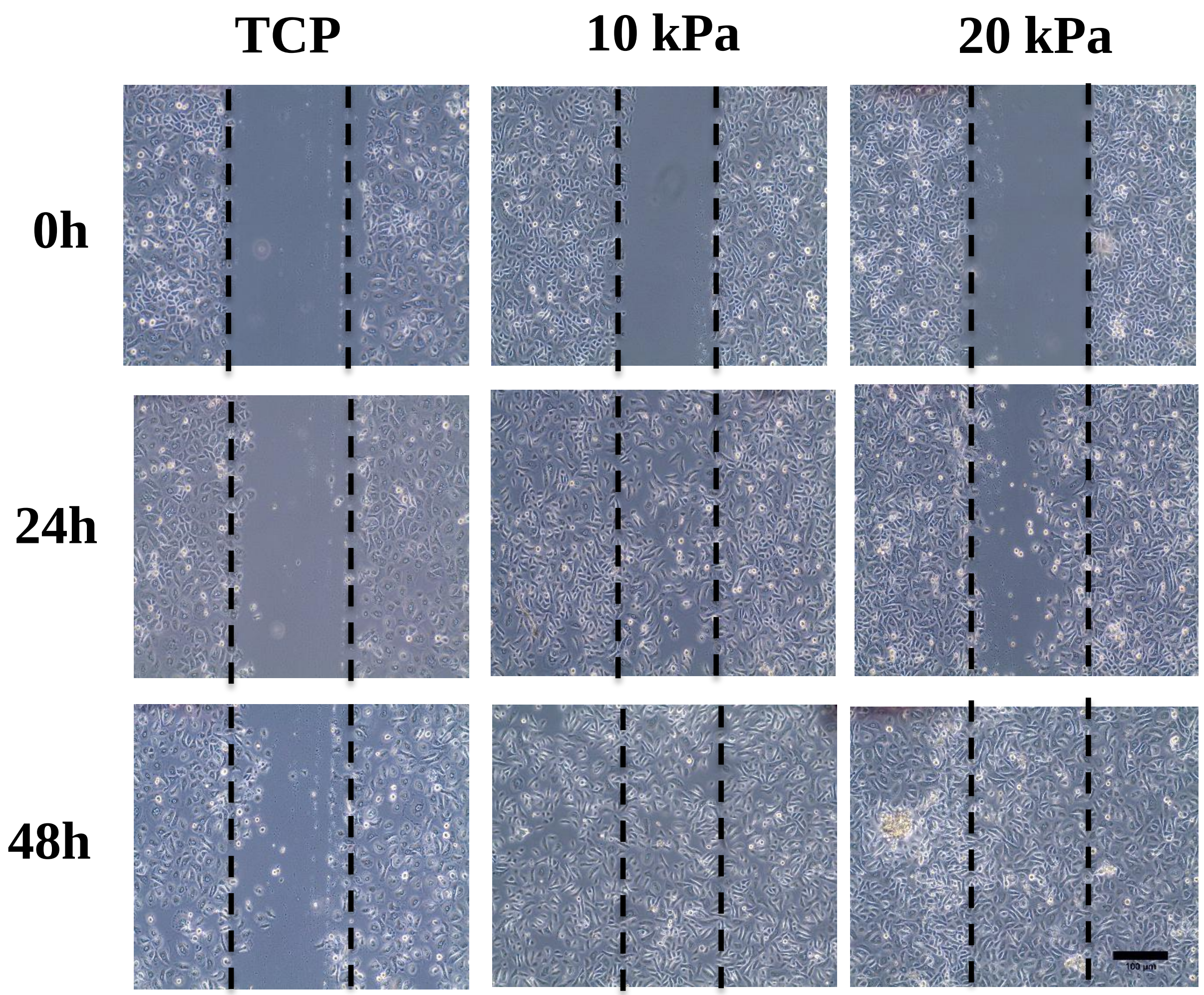

Supplementary Figure 3: Representative images of wound closure by HPKs obtained from TCP, 10kPa and 20kPa of P-8 in the presence of EGF. The black dashed line marks the wound front, beyond which the cell migration is shown till 48h down the column. In the presence of EGF, complete wound closure was seen in 48h by cells obtained from 20 kPa. The cells from 10kPa also retained migratory tendency at P-8, unlike TCP.

Supplementary Figure 3
